# Improved Fracture Healing in Patients with Concomitant Traumatic Brain Injury: Proven or Not?

**DOI:** 10.1155/2015/204842

**Published:** 2015-03-22

**Authors:** Martijn Hofman, Guido Koopmans, Philipp Kobbe, Martijn Poeze, Hagen Andruszkow, Peter R. G. Brink, Hans-Christoph Pape

**Affiliations:** ^1^Department of Orthopaedic Trauma, University of Aachen Medical Center, Pauwelsstrasse 30, 52074 Aachen, Germany; ^2^Algiax Pharmaceuticals GmbH, Max-Planck-Strasse 15a, 40699 Erkrath, Germany; ^3^Department of Trauma Surgery, Maastricht University Medical Center, P.O. Box 5800, 6202 AZ Maastricht, Netherlands

## Abstract

Over the last 3 decades, scientific evidence advocates an association between traumatic brain injury (TBI) and accelerated fracture healing. Multiple clinical and preclinical studies have shown an enhanced callus formation and an increased callus volume in patients, respectively, rats with concomitant TBI. Over time, different substances (cytokines, hormones, etc.) were in focus to elucidate the relationship between TBI and fracture healing. Until now, the mechanism behind this relationship is not fully clarified and a consensus on which substance plays the key role could not be attained in the literature. In this review, we will give an overview of current concepts and opinions on this topic published in the last decade and both clinical and pathophysiological theories will be discussed.

## 1. Introduction

### 1.1. Outline of the Review

In the last 3 decades, there are numerous studies published that either support or reject the hypothesis of enhanced callus development and fracture healing in patients with concomitant traumatic brain injury (TBI). Research on the development of heterotopic ossifications in paralytic patients goes back even further. The first studies on this subject, with the question whether fracture healing is influenced by accompanying TBI, were published in the early 1960s. Despite this history of studies, there is still no hard proof whether there is a relationship between TBI and enhanced callus formation. Moreover, the pathophysiological background of these phenomena is not clarified in the literature. A first review on these subjects was published by Morley and colleagues in 2005 [1]. They reviewed the literature on this topic until 2001, but they did not find a definite answer to their main question if traumatic brain injury results in accelerated fracture healing. The aim of our review is to evaluate the current status of knowledge and to compile an update on this topic. Evidence of a relationship between TBI and fracture healing could be important as a basis for further research to clarify the mechanism of normal and pathologic fracture healing.

## 2. Methods

The following criteria were used to determine eligibility of a study to be included in this review. A literature search was carried out on Medline, Embase, and Cochrane for studies published from January 2001 till December 2012 on the topic of fracture healing in subjects with concomitant traumatic brain injury. The following search terms were used in different combinations: “head trauma,” “brain injury,” “cerebral injury,” “fracture healing,” “bone healing,” “pseudoarthrosis,” and “peri-articular ossifications.” The search was limited to manuscripts in English, German, or Dutch language. Letters to the editor and case reports were excluded. The references of selected studies were also pursued for articles that may have been missed via the electronic search.

### 2.1. Study Selection

The title and abstract of all identified studies (*n* = 2880) were examined by one reviewer (Martijn Hofman). Then, the entire article was obtained and assessed for suitability by two of the authors (Martijn Hofman and Philipp Kobbe). Any issue pertaining to eligibility of studies was solved via discussion with the senior author (Hans-Christoph Pape). This resulted in 26 relevant articles, which were not included in the review of Morley and colleagues. Thirteen articles described clinical studies, of which 6 were prospective and 7 were retrospective cohort studies. An additional thirteen studies were preclinical (“in vitro”/“in vivo”) studies, including one review.

## 3. Fracture Healing

**Figure 1:**
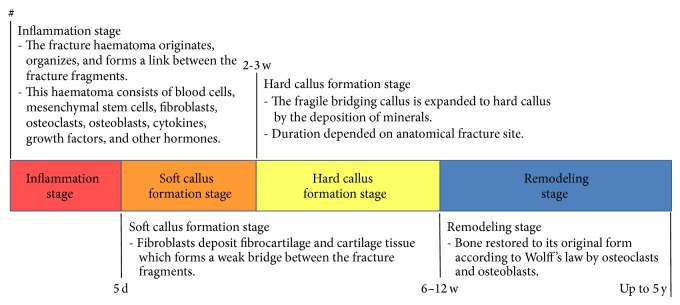
Indirect fracture healing.

Fracture healing occurs either by direct intramembranous healing or by indirect intramembranous and endochondral healing. Indirect fracture healing is the most common form and can be subdivided into multiple stages (Figure 1). The first stage, named the* inflammation stage*, starts with fracture and can last for about 5 days. In this stage, the fracture haematoma organizes and forms a link between the fracture fragments. This haematoma consists of blood cells, mesenchymal stem cells, fibroblasts, osteoclasts, osteoblasts, cytokines, growth factors, and other hormones.

The second stage is called* soft (or bridging) callus formation stage* and lasts for 2-3 weeks. Fibroblasts within the granulation haematoma deposit fibrocartilage and cartilage tissue which forms a weak bridge between the fracture fragments. The duration of the third stage of fracture healing, the* hard (or medullary) callus formation stage*, depends on the anatomical fracture site and will take between 6 and 12 weeks. In this stage, the fragile bridging callus is expanded to hard callus by the deposition of minerals. In the last* stage of remodeling*, which can last up to 5 years after injury, bone adaptation according to Wolff's law occurs. This law explains that, as a response to external loading, bone will be restored to its original form, involving bone resorption by osteoclasts and the formation of new bone by osteoblasts.

In the acute inflammatory reaction of fracture healing cytokines, such as tumor necrosis factor-*α* (TNF-*α*), interleukin-1 (IL-1), and interleukin-6 (IL-6), are involved. TNF-*α* acts as a proinflammatory mediator and a chemotactic agent. Furthermore, it enhances the osteogenic differentiation of mesenchymal stem cells (MSCs) [2]. TNF-*α* peaks at about 24 h and returns to baseline at about 72 h after injury [3]. IL-1 is produced in the acute phase by macrophages in a biphasic mode. IL-1 induces the IL-6-production by osteoblasts, the forming of cartilaginous callus, and the angiogenesis [2, 4, 5].

IL-6 which is only active in the acute phase enhances also angiogenesis, the vascular endothelial growth factor- (VEGF-) production and osteoblasts and clasts differentiation [6].

After traumatic brain injury, the cytokine levels rise both in the cerebrospinal fluid and in the serum. Although the group of Kossmann found an approximately 10 to 100 times higher level of posttraumatic IL-6 and IL-8 levels in the cerebrospinal fluid opposed to the plasma [7–10], it is not clear whether this gradient is caused by a quick peripheral metabolism in the liver [11] or by an initially higher local production in microglia, astrocytes, and macrophages [10, 12]. Furthermore, there are no studies finding an evidence of a direct correlation between the increased levels of cytokines and enhanced fracture healing or callus formation.

Indispensable for fracture healing is the recruitment of skeletal stem cells from surrounding tissues to the fracture site. Skeletal stem cells are mesenchymal stem cells which can differentiate to skeletal cell types including osteoblasts, chondrocytes, adipocytes, fibroblasts, and adventitional reticular cells [13]. These skeletal stem cells have three main functions, that is, they function as signaling centers, they provide a supportive microenvironment for hematopoiesis, and they stabilize and maintain the sinusoidal network within a fracture site. Although the mechanism of recruitment of these cells is still unclear, the recent opinion is that the stromal cell-derived factor-1 and its G-protein-coupled receptor CXCR-4 axis (SDF-1/CXCR-4-axis) play an important role [14–16]. Other concepts of recruitment impute a role to transforming growth factor-*β* (TGF-*β*), bone morphogenetic proteins (BMPs), insulin-like growth factor-1 (IGF-1), cellular retinol-binding protein 1 (CRBP-1), osteoblast stimulating factor (OSF-1), and hypoxia inducible factor-1*α* (HIF-1*α*) [10].

Following activities of the MSCs at the fracture site, another immunological cascade occurs, in which the transforming growth factor-*β* (TGF-*β*) superfamily, especially TGF-*β*2, TGF-*β*3, GDF-5, BMP-5, and BMP-6, seems to be involved [17, 18]. TGF-*β* stimulates growth of cells of the osteoblastic lineage and acts as a chemoattractant for osteoblasts. Another supposed function of TGF-*β* is to increase the endogenous production of morphogens, such as bone morphogenetic proteins [19]. As members of the TGF-*β* superfamily, three members of the BMP subfamily enhance bone growth in peripheral locations, that is, BMP-2, BMP-4, and OP-1 (formerly BMP-7) [20–22]. The work of Spector and colleagues shows that these three BMPs are expressed in early as well as advanced stages of bone healing and remodeling and as soon as mature bone has formed, the concentration of the BMPs normalize again [23].

The revascularization of the fracture and callus site is regulated by the angiopoietin-dependent pathway in which the first vascular ingrowth occurs from existing vessels from the periosteum. However, the main part of the revascularization is regulated by the VEGF pathway, which transforms the cartilaginous avascular matrix into vascularized osseous tissue [24]. In the transformation of soft into hard callus, the Wnt-protein family modulates the differentiation of MSCs into the osteoblastic lineage and later on the osteoblastic bone formation. Another cascade of immune factors, such as macrophage colony-stimulating factor (M-CSF), receptor activator of nuclear factor kappa B ligand (RANKL), osteoprotegerin (OPG), and TNF-*α*, starts the resorption of cartilage and the conversion in calcified bone tissue [25].

The last remodeling stage of fracture healing is regulated by IL-1, TNF-*α*, and some BMPs, especially BMP-2, and by the pressure applied to a crystalline environment [26].

## 4. Traumatic Brain Injury

The prognosis of traumatic brain injury depends on both the primary and secondary brain damage. At the time of the initial traumatic impact on neurocranium and brain tissue, the primary brain injury originates and consists of concussion, contusion, shear injuries, lacerations, and axonal stretching [27]. In cases of severe primary brain injury, in which lesions of neurons, axons, and microglia cells occur, mortality rate is very high.

Subsequent to the primary injury, a delayed complex immunological, biochemical, and physiological pathomechanism, which continues for several days to weeks, results in secondary brain damage [27–29]. This secondary brain damage is a multifactorial process which is caused and influenced by different processes, such as excitotoxicity, inflammation, edema, cell death, mitochondrial damage, magnesium depletion, the production of free radicals, and damage to the blood brain barrier [27, 28, 30].

### 4.1. Excitotoxicity

After primary and secondary brain injury, excitotoxicity derives from the breakdown of neurons loaded with excitative neurotransmitters [28, 31]. Of these released neurotransmitters, glutamate is the most prominent neurotransmitter throughout the brain. This secretion of glutamate starts several minutes after the primary trauma, peaks about 10 minutes after trauma, and stays increased for several days [31]. Through this glutamate discharge, an autodestructive cascade is initiated by way of a calcium influx followed by a calcium overload, resulting in a stimulation of calcium-dependent enzymes, such as proteases, lipases, and endonucleases [27].

### 4.2. Inflammation

Generally after trauma, classical or neurogenic, an inflammation cascade follows and the immune system is dysregulated, which influences the neurologic injury negatively [28]. Cytotoxic and inflammatory events with infiltration of leukocytes, macrophages, lymphocytes, and natural killer cells will occur. There are a lot of mediators, which influence the inflammation process after TBI. Amongst others, these are complement components, chemokines, and cytokines [32].

The complement system is upregulated after TBI by passive leakage across the damaged BBB or by intracerebral synthesis [33–35]. As a “first line of defence,” this system promotes the inflammation by recruiting proinflammatory molecules, phagocytosis, apoptosis, and damaging of the BBB.

Other important proinflammatory modulators are chemokines. These heparin-binding proteins, which are produced by inflammatory cells, promote the infiltration of leukocytes in the traumatized brain tissue and thereby enhance the inflammatory reaction after TBI.

There are also many cytokines which have a proinflammatory role in the process after TBI. By the release of neuropeptides from sensory neurons, in case of neurogenic inflammation, extravasation of plasma, vasodilatation, and neuronal hypersensitivity results [36], the most prominent neuropeptides involved are members of the bradykinin- and tachykinin-family of which CGRP (calcitonin gene-related peptide) and substance P are the most distinguished exponents [37].

However, several studies in the last decade impute a dual role for some mediators supposing a proinflammatory and an anti-inflammatory effect in the post-TBI inflammatory process. This dual role is often demonstrated by a time-dependent pro- and anti-inflammatory characteristic of the different immune modulators, such as IL-1, IL-6, TNF-*α*, and chemokine (especially fractalkine (CX_3_CL_1_)) [38–41].

For TNF-*α*, which appears to be synthetized in the brain tissue itself, as a endogenous response to TBI in the first few hours, such a dual function is demonstrated in several studies [39, 41–43]. These studies demonstrate an early proinflammatory (1-2 d) and a late anti-inflammatory (2–4 wk) role for TNF-*α*. TNF-*α* increases vascular permeability leading to swelling of brain tissue and leukocyte infiltration. It also induces necrosis and apoptosis via intracellular pathways and it upregulates the inflammatory mediator anaphylatoxin (C5a) on neurons [44].

An important role in the inflammation after TBI is reserved for the interleukin-1-family. IL-1 induces neuronal apoptosis and VEGF, an important mediator for the generation of posttraumatic oedema. The exact effect of IL-1, neurotoxic or neuroprotective, depends on the environment in which this cytokine resides. Also, IL-18 has raised concentrations in the cerebrospinal fluid after TBI [45] and by inhibiting both IL-1 and IL-18, the secondary brain damage after TBI could be reduced [41]. The dual role of the interleukin-family shows a proinflammatory phase in the first hours and days after TBI followed by a reparative phase lasting for days to months [46].

Also IL-6, which is produced by neurons and macrophages early after TBI (1 h after injury), can promote the inflammatory response but also has anti-inflammatory effects [42, 43]. These anti-inflammatory effects are increased by the capacity of IL-6 to inhibit TNF-*α* synthesis, induce nerve growth factor (NGF), promote survival and differentiation of neurons, and antagonize* N*-methyl-D-aspartate-mediated toxicity [42]. Another aspect of IL-6, as a VEGF-agonist, is its quality to enhance angiogenesis and revascularization and thereby promote brain tissue repair [47–50].

TGF-*β*, which increases within the first days after injury, may be produced by virtually all cells of the central nervous system (CNS). This growth factor counteracts the inflammation process by suppressing the release of IL-1, TNF-*α*, IFN-*γ* (interferon-*γ*), oxygen radicals, MHC class II antigen expression, T-cell activation, and proliferation of various cells [51–55]. On the contrary, the chemotactic function of TGF-*β* leads to leukocyte invasion and deposition of extracellular matrix (ECM) and scar tissue formation. These latter functions are more proinflammatory [56, 57].

### 4.3. Edema

Although many factors contribute to the morbidity and mortality, the extent of cerebral edema seems to be the supreme predictor of functional outcome after TBI [37, 41, 58]. There are two forms of edema identified after TBI. The vasogenic edema, which is caused by the extravasation of fluid from the vasculature, has an early onset after trauma and is associated with an increased permeability of the BBB. The subsequent cytotoxic edema originates from an osmotic shift of extracellular fluid to the intracellular compartment. The latter forms as neurotoxic qualities and accounts for most of the brains welling after TBI [37, 59]. Key players in the development of postinjural edema are aquaporins (AQPs), matrix metalloproteinases (MMPs), and vasoactive agents. The expression of several AQPs, which are integral membrane proteins, is upregulated after TBI and promotes edema formation [60]. The MMPs are zinc-dependent endopeptidases involved in the process of tissue remodeling following various pathologic conditions. The regulation of the MMP expression is complex and in cases of dysregulation by TBI, stroke, or neurodegeneration synaptic loss and breakdown of the BBB is identified, causing a vasogenic edema and subsequent cell death [61–64]. The most important vasoactive agents are members of the bradykinin- and tachykinin-families and are produced in the neurogenic inflammation process. In particular, substance P is thought to enhance edema formation [37].

In case of posttraumatic edema formation, the swelling of cells as well as parenchyma swells and leads to an elevation of the intracranial pressure (ICP), with a subsequential decrease of cerebral perfusion pressure (CPP). Eventually a herniation of the brain stem can occur [37].

### 4.4. Cell Death

Cell death after TBI occurs in the first 24 h foremost via necrosis, in which swelling of mitochondria and other organelles and subsequent membrane degeneration occurs. In the subsequent days, cell death occurs also via apoptosis, in which DNA condensation and fragmentation, cell shrinkage, and the ultimate formation of apoptotic bodies occur [65]. Another complicating factor is that the inhibition of one mechanism of cell death can exacerbate the other mechanism of cell death and vice versa [66, 67].

Furthermore, beside the release of multiple proinflammatory modulators by the neurogenic inflammation cascade as described above, apoptotic and necrotic cells will set free multiple cytotoxic cytokines, growth factors, and interleukins, which will lead to a vicious circle of inflammation and cell death, which can last for months after the initial trauma [41, 42, 68].

### 4.5. Mitochondrial Damage

Brain and nerve tissues have a high energy demand and therefore the mitochondria are of utmost importance for the survival of these tissues. Sever injuries to the mitochondria can elicit devastating alterations to the mitochondrial respiration, respiratory coupling, and energy production [69–71].

### 4.6. Magnesium Depletion

A universal aspect of central nervous system (CNS) injury is a decrease of intracellular free magnesium, which plays normally a crucial role in normal cell function by regulating numerous physiological and biochemical processes within the cell [72]. Magnesium is a required cofactor in all energy producing and consuming reactions and over 300 enzymes involved in these processes are magnesium dependent [27]. Beside these effects on enzymes, plasma membrane integrity and ion channel activity are also influenced by magnesium [73].

### 4.7. Production of Free Radicals

In the oxidative metabolism, free radicals are produced as normal by-products. The production of these highly reactive molecules is significantly enhanced by traumatic injuries [74–76]. Proteins, DNA, and lipids can be damaged by high concentrations of these free radicals, which lead to cell death via apoptosis [77].

### 4.8. Blood-Brain-Barrier Damage

If a cerebral mediator is released by TBI, which influences bone healing, it has to cross the blood brain barrier (BBB). The BBB is formed by the neurovascular unit, a conjunction of cerebrovascular endothelial cells, pericytes, astrocytes, and the basal lamina [78]. The BBB strongly regulates the exchange of substances between plasma and the cerebral interstitium [32]. After TBI, there occurs a biphasic BBB disruption, with hyperpermeability in the beginning with a maximum at 4–6 h after injury, followed by a transient restoration and a prolonged period of hyperpermeability [37, 79, 80]. Both small and large molecules are able to cross this barrier in and around the injury site [81]. The restoration of the BBB lasts from about 4 hours for large molecules to about 4–7 days for small molecules [37, 81]. There are many factors influencing the permeability of the BBB. In the beginning, the disruption is caused by a mechanical force, but in the later course of TBI other mediators are responsible for influencing the BBB, such as VEGF, angiopoietins, IL-1*β*, IL-8, TNF-*α*, reactive oxygen species, kinins, histamines, nitric oxide, elastase, and matrix metalloproteinase (MMP) [82–90]. Furthermore, this permeability is also influenced by hypoxia after TBI, mostly after about 6 h after injury and it can delay the restoration of the BBB by up to 72 h [91].

## 5. Review of the Literature

When we consider all studies on the topic of TBI and bone healing in the last decades, more than 50 different cells, hormones, growth factors, cytokines, chemokines, and so forth are reviewed and in a few cases there are some promising results (Table 1).

In the review of Morley and colleagues, they conclude that there is evidence for an accelerated osteogenesis associated with TBI. They found a relationship between TBI, rapid callus development, and stimulation of bone forming cells [1, 99, 100]. However, they could not differentiate between heterotopic ossification and accelerated fracture healing [1].

In the last decade, there were 26 studies on this topic after the publication of Morley and colleagues. About 50% of these studies [92–98, 101–107] find evidence for increased callus formation in cases with concomitant TBI; the other studies are not conclusive.

There are only 7 of these studies which also postulate a possible working mechanism for this correlation. These are the studies we focus on in our review (Table 2).

### 5.1. Human Mesenchymal Stem Cells (hMSCs)

Kanczler and Oreffo stress the importance of angiogenesis in combination with osteogenesis to optimize bone growth [108]. In fracture healing angiogenesis precedes osteogenesis. Xiao and colleagues show that bone marrow stromal stem cells (BMSCs) have the possibility to express both BMP-2 and VEGF and so these mesenchymal stem cells enhance fracture healing more explicitly compared to the addition of any single factor [109]. Also Yamada and colleagues confirm the importance of a combination of osteogenic and angiogenic factors in the regeneration of bone. They found that a mixture of platelet-rich-plasma and mesenchymal stem cells can elicit better bone regeneration with good vascularization [110]. In the cascade after fractures, mesenchymal stem cells play an important role. These MSCs originate from bone marrow, periosteum, and so forth and are in case of a fracture generated to migrate to the fracture site in response to BMPs set free from the injured bone matrix [111, 112].

At the fracture site these MSCs produce different proteins and these proteins can differentiate the mesenchymal stem cells at their turn to enhance the fracture healing process [23].

As multipotent cells, the MSCs can differentiate into different mesenchymal lineages which support the formation of distinct tissues, such as bone, cartilage, fat, tendon, muscle, and bone marrow stroma [110, 113].

This differentiation takes place under influence of different factors. Beside the proteins coming from the mesenchymal stem cells as described above, Boes and colleagues propose an influence of unknown factors released by injured brain tissue, which exert their proliferative effect specific to mesenchymal stem cells [92]. In their in vitro analysis, they showed that the serum of rats with a fracture and concomitant TBI stimulated a multipotent mesenchymal stem cell line (C3H10T-cells) to proliferate at a significantly higher level (*P* = 0.0002), resulting in a 76% increase in cells in the fracture/TBI group compared to the fracture-only group. They also investigated an osteoblastic (MC3T3-14-cells) and a fibroblastic (NIH 3T3-cells) cell line, but here they did not find any difference in proliferation rate [92]. Boes and colleagues compared the callus in rats with a femur fracture with concomitant TBI and without concomitant TBI. It was shown that after 21 days, the callus in the TBI and fracture group was reduced in diameter (*P* = 0.030), but it is significantly stiffer (0.306 N/mm compared with 0.120 N/mm; *P* = 0.02) than the fracture-only group. The torsional strength was equal in both groups (258.4 Nm compared with 231.4 Nm; *P* = 0.472) [92].

The group of Cadosch and Gautschi investigated a human fetal osteoblastic mesenchymal stem cell line (hFOB1.19 cells) in an early stage of its differentiation [94, 95]. In an earlier study, they saw that the cerebrospinal fluid of patients with a traumatic brain injury had an osteoinductive potential and therefore they expected that any osteoinductive factor in the serum of patients with a traumatic brain injury would have an stimulating effect on the hFOB1.19 cells in vitro [114, 115]. This potential reaches its maximum as soon as 6 h after injury it remains at the same level for about 3 days and decreases after about 1 week [94]. They also observed an increased proliferation rate of osteoblasts exposed to sera from patients with TBI during the first week after injury [94].

The time window of this effect is possibly explained by the traumatic disrupture of the blood brain barrier, permitting leakage of cerebrospinal fluid and the recovery of the blood brain barrier after about 1 week. Another explanation for the decreased osteoinductive potential after 1 week is a decreased production of osteogenic factors by the injured brain [94, 95].

Another result reported by the group of Cadosch and Gautschi was an increased expression of the osteoblastic differentiation marker gene in the serum of brain injured patients, such as ALP, CATK, RUNX-2, macrophage colony-stimulating factor, and SP-7 [95, 116, 117].

In the clinical part of their research, Gautschi and colleagues observed, in the TBI and fracture group in 41.7% of the cases, clinical and radiological evidence for hypertrophic callus formation. None of the cases in the fracture-only group developed hypertrophic callus. Moreover, Cadosch and colleagues found a positive correlation between callus ratio and proliferation of hFOB1.19 cells. On the contrary, they found an inverse correlation between fracture union time and callus ratio as well as between Glasgow Coma Scale (GCS) and callus ratio [95].

In a recent in vitro study of Yang and colleagues increased levels of arachidonic acid (AA) were noted in serum metabolites of rats after TBI. They showed that in the presence of arachidonic acid the expression and proliferation of bone gamma carboxyglutamate protein (BGLAP or osteocalcin) is beneficially influenced and thereby the proliferation of the mouse osteoblastic cell line MC3T3-E1 was increased. They suggest a key role for arachidonic acid in the process of enhanced callus formation in rats with a TBI [98].

### 5.2. Leptin and CGRP

In the metabolic, inflammatory, and neuroendocrine stress response occurring after TBI serum levels of leptin, an adipose-derived hormone, are significantly increased [93]. The level of leptin is further influenced by different cytokines and hormonal factors, but the exact pathway of how leptin influences bone formation is not fully understood. It is postulated that the proinflammatory cytokine IL-1 increases rapidly after brain injury and this might cause an increased serum level of leptin [118, 119].

Moreover, Wei and colleagues [93] found a positive correlation between leptin concentration in serum and volume of callus formation in patients with a fracture and a traumatic brain injury. Leptin can influence bone metabolism by two pathways. In the first central pathway, leptin exerts its antiosteogenic effect via an increased sympathetic output controlled by the hypothalamus. In the second peripheral pathway, leptin has the opposite effect and promotes bone mineralization and osteoblast-to-osteocyte differentiation [93]. They suggested several tracts for the serum level increase of leptin. First of all, the mobilization of free fatty acids as a result of hypermetabolism in traumatic brain injury patients results in elevated serum leptin levels by a neuroendocrine feedback mechanism [120]. Secondly, hypoxia, caused by the adult respiratory distress syndrome or a pulmonary inflammatory response in traumatized patients, augments adipocyte expression of leptin [121]. Finally, the release of bone marrow at the fracture ends, containing mainly hematopoietic cells, induces leptin delivery at tissue level [122]. Furthermore, the complex neuroendocrine inflammatory response after TBI, with the release of multiple cytokines and hormones, influences the production and levels of leptin.

Wei and colleagues have shown that the serum leptin concentration reaches a significant increased level only from the 4th until the 12th week after injury. This can be explained by the fact that in the acute posttraumatic period the stress response increases the sympathetic outflow, which downregulates leptin expression and secretion by adipocytes. The secretion of leptin by adipocytes is also decreased due to the fasting state of a trauma patient in the acute posttraumatic stage. After the initial posttraumatic period, the peripheral effect of increasing leptin may outweigh the sympathetic inhibition of leptin on bone formation [123, 124].

The results of Wei and colleagues confirm the concept of Rayner and Trayhurn in 2001 and of Takeda and colleagues in 2002 that injury to the hypothalamus may result in additional peripheral secretion of leptin, which in turn may impair the antiosteogenic effects of leptin through the loss of sympathetic leptin inhibition, in this way contributing to the enhanced bone regeneration observed in rats with fractures and TBI [93].

In their evaluation of callus formation, Wei and colleagues show a significantly increased callus volume in rats with TBI and fracture compared to rats with only a fracture from the 4th till the 8th week after injury. This increase subsides at 12 weeks after injury, but in the histological analysis they can still proof thicker lamellar bone formation in the TBI and fracture group at 12 weeks after injury [93].

Another possible mode of action of leptin on fracture healing is via calcitonin gene-related peptide (CGRP). Zhang and colleagues [125] showed in 2011 that peripheral administration of leptin alleviated injury-evoked brain damage by promoting CGRP expression, improving regional cerebral blood flow, and reducing local infarct volume and neurological deficits. Furthermore, leptin also promoted bcl-2 expression and suppressed caspase-3 in vivo and vitro after injury. Administration of CGRP(8-37), an antagonist of the CGRP receptor, partly abolished the beneficial effects of leptin and restored the normal expression levels of bcl-2 and caspase-3 in neurons, which indicated that leptin-induced protection of neurons was correlated with release of CGRP.

Results of Song and colleagues showed in vivo a significantly elevated concentration of CGRP in rats with a femoral fracture and a concomitant traumatic brain injury. These concentrations were found in both the brain tissue and muscle tissue surrounding the fracture. They concluded that there was reciprocity between traumatic brain injury and enhanced fracture healing and they suppose that the CGRP is produced by the brain tissue. This conclusion was based on the observation that CGRP was expressed in the cerebral cortex and around the fracture site in the TBI and fracture group and not in the fracture-only group [97].

In their micro-CT analysis of callus formation, they observed a significantly increased bone mineral density (BMD) and bone mineral content (BMC) of the newly formed callus in the TBI and fracture group compared to the fracture-only group in the 4th week after injury. The same measurement in the 8th week after injury revealed that the BMD was indifferent and the BMC was significantly lower in the fracture-only group when compared to the TBI and fracture group. According to Song and colleagues, this indicated that fracture healing occurs earlier in cases with concomitant TBI [97].

In 2000 García-Castellano and colleagues [126] already confirmed a great effect of CGRP on angioectasia to capillary, which is, as discussed before, of extreme importance for the acceleration of bone formation. According to Zhang and colleagues [96] CGRP can exert this hemangiectasic role at the fracture site due to an axoplasmic transport of CGRP from the central nerve system.

## 6. Discussion

For years physicians declare, according to their clinical experience, that callus formation/heterotopic ossification and fracture healing are accelerated in patients with accompanying traumatic brain injury. However, these statements are not based on hard evidence according to the literature, because the level of evidence in all of these clinical studies is not very high.

Before the review of Morley et al. [1] in 2005, there were only 5 clinical studies which found proof of enhanced callus formation in patients with TBI. These studies included one prospective study [99] and 4 retrospective studies [100, 127–129].

In the last decade, six clinical studies were published, which found proof of an accelerated callus formation or fracture healing in patient with TBI and concomitant fractures. Five of them were prospective matched-control studies [94, 95, 98, 102, 103] with an average patient population of 65.2 (range 28–86) patients and one study was a retrospective, matched-control study [101], with a patient population of 67 patients.

In the period before 2003, there were 4 clinical studies which objected to this clinical experience because these studies did not find significant differences in callus formation or fracture healing between patients with and without traumatic brain injury. Of these 4 studies, there is only one prospective matched-control study [130], but with 8 patients which is rather a case series. The other 3 studies are retrospective studies [131–133], of which only one study is matched-control.

In the last decade, there are no studies published which refute this enhanced callus formation in patients with concomitant TBI, but here publication bias must be considered.

When we consider the studies which demonstrate a mechanism of action for the enhanced callus formation in patients with concomitant TBI, there are two mainstreams in the last decade.

The first mainstream is represented in 4 [92, 94, 95, 98] of the 7 considered articles and puts the human mesenchymal stem cells/skeletal stem cells in focus.

In the study of Boes et al. [92], adult rats were investigated, which were 7–9 months of age. The traumatic brain injury was administered when the animals showed signs of normal recovery after the administered femoral fracture and subsequent osteosynthesis, but the timing between administering the two lesions was not mentioned. The fact that the TBI and the fracture are not administered at the same time, as is the case in normal trauma patients, could be a confounding factor.

The biomechanical analysis and in vitro analysis of cell proliferation were both performed at 21 days after injury. This time point is very early because it is known that full recovery of mechanical properties of fractured femora in rats takes about 4 weeks in young and about 12 weeks in adult rats [134]. Nevertheless, they already found a significant increased stiffness of the fracture site in the fracture and TBI group compared to the fracture-only group. This probably could be even more distinct in a later stage of fracture healing. Boes et al. [92] conclude that the reduced callus diameter in the fracture and TBI group contradicts the concept that TBI increases endochondral ossification and they suggest that the fractures already have progressed into the remodeling phase. Regarding the early time point at which these investigations are performed, this does not, in our opinion, contradict the above-mentioned concept because fracture healing has not finished yet and the remodeling phase probably has not started yet.

Also, the increase in the proliferation of the mesenchymal stem cell line C3H10T_1/2_ of 76% is an impressive result of the study group, which attributes this enhancement to a yet unknown soluble factor from injured neural tissue. Although the group of Boes et al. [92] could not show an increased proliferation of a more advanced osteoblastic cell line (MC3T3-14), the group of Yang et al. did observe an increased proliferation of a more differentiated osteoblastic cell line of MC3T3-E1 cells. Of note, the analysis was performed with serum of TBI rats which was taken only at one time point, that is, 24 h after injury. In the bone healing process, the first few days are mainly determined by the inflammation phase in which the osteoblasts maybe play a subordinate role.

The results of the clinical part of the study of Gautschi et al. [94] are convincing with >40% of hypertrophic callus formation in the TBI and fracture group in comparison with the fracture-only group, although the study groups are considerable small (resp. *n* = 12 and *n* = 19). They refer to preclinical studies of Boes et al. [92], Mandelin et al. [116], and Camozzi et al. [117] in which proof is found for brain-derived factors with mitogenic and osteogenic effects on stromal stem cells and molecular mechanisms of sera from brain injured patients which mediate a mitogenic effect on osteoprogenitor cells. Cadosch et al. support these findings because they find a negative linear relationship between GCS and callus ratio on one site and time to union and callus ratio on the other site [95]. The proof of decreased time to union in patients with concomitant TBI is decisive in contrast with many other studies because time to union was an endpoint in their study and it was determined by two independent blinded radiologists.

In their cell proliferation assay with primary human osteoblasts, they harvested the osteoblasts from 20 patients in which an osteosynthesis was performed. At what time these osteosyntheses were performed and from which group these samples were taken are not described. As a consequence, it remains unclear whether these osteoblasts were already stimulated in the body of the patients before harvesting. They also show that the osteoinductive effect of serum from TBI-patients on the hFOB-cell line is increased from 6 h after injury until 3 days after injury. This effect fades after about 1 week. This could mean that this effect functions as a trigger for an enhanced callus formation. Whether this callus formation starts earlier through this trigger or evolves at a greater speed is still unsolved.

In the other study of Cadosch et al. [104], the increased proliferation lasts even longer until the last measurement at 168 h after injury.

Because the observation period in both studies only lasted for 1 week after injury, it is unclear if this osteoinductive effect returns to normal levels after this week or that it has an analogous fluctuation as several cytokines in their dual function, with an additional effect later on in the fracture healing process. The fact that the disruption of the BBB lasts for about one week and therefore the leakage of influencing factors produced by injured brain tissue will last for this period could be supportive to the findings of Gautschi and Cadosch [94, 95, 104].

Gautschi et al. [94] show an interesting pattern in the expression of osteoblastic markers (alkaline phosphatase (ALP), runt-related transcription factor 2 (RUNX-2), cathepsin K (CATK), and serine protease 7 (SP 7)), measured by the expression of mRNA in hFOB cells. ALP and RUNX-2 expression is significantly increased in the TBI and TBI and fracture group compared to the fracture-only group and the expression of SP 7 and CATK is significantly increased only in the TBI and fracture group compared to the TBI and the fracture-only group. This could mean that the expression of SP 7 and CATK is even more specific to the relationship between TBI and enhanced fracture healing than the expression of ALP and RUNX.

Furthermore, the fact that the results of the publications from Gautschi and Cadosch [94, 95, 104] are very similar should be put in the perspective that both studies come from the same group of researchers.

The second mainstream is represented by Wei et al. [93], Zhang et al. [96], and Yang et al. [106] and they focus on the role of leptin and CGRP in the healing of fractures in patients with TBI.

In the study of Wei et al. [93], which links serum leptin levels and leptin expression in callus cells to increased callus formation, they suggest a relationship between hypothalamic damage and reduced inhibition of peripheral leptin secretion and increased callus formation. This increase in leptin levels is reached only after 4 weeks after injury. In contrast to the studies on mesenchymal stem cells of Cadosch and Gautschi [94, 95, 104], Wei et al. [93] extended the time slot of their histomorphological and histochemical analysis until 12 weeks after trauma. Considering the fracture healing process in adult rats, this is better suitable than a period of only 1 week.

Zhang et al. [96] showed the link between increased leptin levels and an enhanced expression of CGRP and its hemangiectasic role at the fracture site.

Song et al. support these results and found proof for a correlation between increased fracture-healing tendency secondary to traumatic brain injury and high CGRP. They found a higher level of CGRP in the brain and muscles of traumatic brain injury rats and they suggest this CGRP is produced by brain tissue [97].

According to their micro-CT analysis of callus formation, Song et al. conclude that in rats with concomitant TBI fracture healing occurs earlier as in the fracture-only group. This earlier healing appears in their population around the 4th week after injury and while these experiments are done on adult rats, this seems almost too early for fully developed callus formation [134].

Another important finding of Zhang et al. is that different types of neural injury affected fracture healing in a different way. That is, peripheral nerve damage in combination with fractures can decelerate the healing process, and central nerve damage in combination with fractures can accelerate the healing rate. This increased healing rate as well as the inflammatory reaction following central neural injury is more intense and distinct for spinal cord injuries than cerebral injuries [68, 96], which could be of importance for future research.

### 6.1. Future Perspectives

The basic proof of enhanced fracture healing in patients with concomitant TBI is not yet substantiated by a large prospective clinical study. If this phenomenon can be proven, the interesting question is whether this mechanism is cellular or hormonally induced.

Important for future research is that the time course of mediator upregulation following TBI will be elucidated as it is done for spinal cord injury by Donnelly and Popovich [68]. As the osteoinductive reaction after spinal cord injury is more pronounced compared to that after TBI, it could be an interesting approach to look at accelerated callus formation in patients with spinal cord injury.

In case of the mesenchymal/skeletal stem cells as well as the hormonal cascade, it is important to look at the fluctuation and influence within the fracture healing process over a longer period than 1 week after injury. Furthermore, the idea that enhanced callus formation in patients with fractures and concomitant TBI is nothing more than a form of heterotopic ossification, which is also supported but not substantiated by several authors and studies in the last decades. This also could be an interesting reference point for further research.

## 7. Conclusions

In 2005 Morley and colleagues concluded that the question whether traumatic brain injury results in accelerated fracture union could not be answered at that time. Today, more than three decades after the first publications on this subject, the consensus in all published papers after 2005 is that traumatic brain injury indeed accelerates fracture healing. However, the greater part of studies on this topic is preclinical (in vitro/in vivo) studies, which indeed find some evidence for certain mechanisms, but the clinical studies with relatively small populations cannot, hitherto, support the hypothesis of accelerated fracture healing in patients with TBI. Moreover, in the last decade, the possibility of publication bias cannot be eliminated.

Furthermore, elucidation of the very complex mechanism of enhanced callus formation in patients with TBI is still in its infancy. Also in the last years there very little is revealed of the complex mechanism by which cytokines, chemokines, hormones, and growth factors influence the signaling pathway leading to accelerated fracture healing.

The studies discussed in this review indicate that both serum and cerebrospinal fluid (CSF) from patients with fractures and concomitant TBI have osteoinductive potential. It also seems to be a consensus in the literature that these osteoinductive factors are released from the injured brain and from their spread in the body and to the fracture region.

In the literature published after the review of Morley and colleagues, there are two mainstreams which impute key roles in the complex mechanism of enhanced callus formation for mesenchymal stem cells on one side and the leptin-CGRP-axis on the other side.

Nevertheless, because callus formation originates from a complex multifactorial cascade, it is possible that all described factors have their role in this phenomenon.

In our opinion, first of all these findings should be an incentive to design a large prospective clinical study to prove or reject the hypothesis of accelerated fracture healing in patients with concomitant TBI. The research on the pathophysiological relationship between TBI and callus formation should also be elaborated to reveal possible pathways of this assumed affiliation.

## Figures and Tables

**Table 1:** Investigated substances.

Cytokines and growth factors		Mesenchymal stem cells	Genes	Hormones	Proteins and enzymes	Others
IGF-1 IGFBP-3 IL-1(*β*) IL-4 IL-5 IL-6 IL-13 TNF-*α* CCL-2 (MCP-1) CCL-20 CXCL1 TGF-*β* BMP2	BMP4 OP-1 (BMP7) rhBMP (−2/−7) VEGF RANKL OPG M-CSF IGF-II PDGF BFGF	hMSC C3H10T1/2-cells MC3T3-cells NIH3T3-cells hFOB cells BMSC PP1-cells PP6-cells	ALP CATK RUNK-2 LacZ	Leptin Corticosteroids Calcitonin CGRP (calcitonin-gene-related peptide) Thyroxin Parathyroid hormone Androgens Growth hormone Prolactin	Alkaline phosphatase Precursor type I collagen CRP Osterix protein (Sp7)	Calcium Phosphate

**Table 2:** Studies with evidence for a certain mechanism of action.

Study	Year of publication	Clinical/preclinical	Number of subjects	Matched control group	Suggested mechanism of action
Boes et al. [92]	2006	Preclinical	*n* = 43	yes	Increased proliferation of **mesenchymal stem cells**, more specifically **C3H10T1/2 cells**, due to brain injury

Wei et al. [93]	2008	Preclinical	*n* = 64	yes	Increased callus formation through an increased **leptin** level at the fracture site

Gautschi et al. [94]	2009	Combined clinical and preclinical	*n* = 61	yes	Increased proliferation and differentiation of **mesenchymal stem cells**, caused by the release of osteoinductive brain-derived factors

Cadosch et al. [95]	2009	Combined clinical and preclinical	*n* = 41	yes	Increased proliferation of the **mesenchymal osteoprogenitor cell line hFOB1.19**

Zhang et al. [96]	2009	Preclinical	*n* = 72	yes	Increased secretion of **calcitonin gene-related peptide** in traumatic brain injury group

Song et al. [97]	2012	Preclinical	*n* = 24	yes	Increased concentration of **calcitonin gene-related peptide** in serum released from injured brain tissue

Yang et al. [98]	2012	Preclinical	*n* = 36	yes	Increased concentration of **arachidonic acid** in serum released from injured brain tissue enhancing BGLAP expression and proliferation of osteoblasts **(MC3T3-E1 cell line)**

## Abbreviations

AAA: Arachidonic acid
BBB: Blood brain barrier
BGLAP: Bone gamma carboxyglutamate protein
BMC: Bone mineral content
BMD: Bone mineral density
BMPs: Bone morphogenetic proteins
BMSCs: Bone marrow stromal stem cells
CGRP: Calcitonin gene-related peptide
CNS: Central nervous system
CRBP-1: Cellular retinol-binding protein 1
CSF: Cerebrospinal fluid
C5a: Anaphylatoxin
d: Days
ECM: Extracellular matrix
GCS: Glasgow Coma Scale
h: Hours
HIF-1 *α*: Hypoxia inducible factor-1*α*
IGF-1: Insulin-like growth factor-1
IL: Interleukin
IL-1: Interleukin-1
IL-6: Interleukin-6
IFN: Interferon
M-CSF: Macrophage colony-stimulating factor
MMPs: Matrix metalloproteinases
MSCs: Mesenchymal stem cells
NGF: Nerve growth factor
OPG: Osteoprotegerin
OSF-1: Osteoblast stimulating factor
RANKL: Receptor activator of nuclear factor kappa B ligand
SDF-1: Stromal cell-derived factor-1
SP-7: Osterix protein
TBI: Traumatic brain injury
TGF-*β*: Transforming growth factor-*β*
TNF-*α*: Tumor necrosis factor-*α*
VEGF: Vascular endothelial growth factor
w: Weeks.
